# Validating the Black Identity, Hair Product Use, and Breast Cancer Scale (BHBS) Among Black Breast Cancer Survivors

**DOI:** 10.3390/ijerph22020174

**Published:** 2025-01-27

**Authors:** Dede K. Teteh-Brooks, Marissa Ericson, Traci N. Bethea, Lenna Dawkins-Moultin, Nicole Sarkaria, Jared Bailey, Adana A. M. Llanos, Susanne Montgomery

**Affiliations:** 1 MD Anderson Cancer Center, Department of Health Disparities Research, University of Texas, Houston, TX 77030, USA; ldawkins@mdanderson.org; 2 Department of Psychology, California Lutheran University, Thousand Oaks, CA 91360, USA; mericson@callutheran.edu; 3 Office of Minority Health & Health Disparities Research, Georgetown Lombardi Comprehensive Cancer Center, Georgetown University, Washington, DC 20003, USA; tb988@georgetown.edu; 4 Department of Health Sciences, Crean College of Health and Behavioral Sciences, Orange Campus, Chapman University, Orange, CA 92866, USA; 5 Gangarosa Department of Environmental Health, Rollins School of Public Health, Emory University, Atlanta, GA 30322, USA; 6 Department of Epidemiology, Mailman School of Public Health, Columbia University Irving Medical Center, New York, NY 10032, USA; al4248@cumc.columbia.edu; 7 Department of Social Work and Social Ecology, School of Behavioral Health, Loma Linda University, Loma Linda, CA 93350, USA; smontgomery@llu.edu

**Keywords:** breast cancer, black identity scale, survivors, black women, BHBS

## Abstract

Personal care products containing toxic chemicals (e.g., endocrine-disrupting chemicals) may increase breast cancer risk, especially for Black women who use these products more than other racial groups. There are limited tools that examine the intersections of identity, behaviors, and attitudes surrounding product use, perceived safety, and breast cancer risk; thus, the Black Identity, Hair Product Use, and Breast Cancer Scale (BHBS) was developed to bridge this gap. While initial validations lacked diverse survivor representation, this study seeks to validate the BHBS among Black survivors. Methods: This study is a part of the *Bench to Community Initiative (BCI)*, where respondents (*n* = 167) completed a 41-item survey including the BHBS between 2020 and 2022. The use of Principal Component Analysis (PCA) and confirmatory factor analysis (CFA) established the underlying component structures and model fit. CFA measures used to confirm component structures included the Root Mean Square Error of Approximation, the Comparative Fit Index, and the Tucker Lewis Index. Results: Black survivors on average were diagnosed with breast cancer before age 40 (37.41 ± 8.8) with Stage 1 (45%) disease. Sixty-three percent of the total variance resulted in a two-component structure. Subscale 1 (S1) measures the sociocultural perspectives about hair and identity (28% of the total variance; α = 0.73; 95% CI = 0.71–0.82). Subscale 2 (S2) can be used to assess perceived breast cancer risk related to hair product use (35% of the total variance; α = 0.86; 95% CI = 0.81–0.94). The two-component structure was confirmed with Root Mean Square Error of Approximation = 0.034, Comparative Fit Index = 0.93, and Tucker Lewis Index = 0.89. Discussion/Conclusions: The BHBS is a valid tool to measure identity, attitudes, and behaviors about product use and breast cancer risk among survivors. Hair is a significant cultural identity expression, and the health effects of styling products should be considered in future interventions.

## 1. Introduction

A Black woman’s hair has been a significant indicator of identity and ancestral roots and may be classified in social environments as professional, natural, or inappropriate [1]. Ideas of Black beauty norms are controlled through policies, media, and interpersonal racism at work, school, or other community settings. Often internalized, westernized perceptions of beauty perpetuate a culture of self-mediated racism that defines the style of one’s hair [2,3]. The CROWN Act—Creating a Respectful and Open World for Natural Hair—was created in 2019 to protect against discrimination of race-based hairstyles, such as braids, locs, and twists, in the workplace and public schools [4]. Despite these policy efforts, Black women with natural hairstyles are still perceived as less professional and less competent and are less likely to be invited for interviews for jobs than White women with curly or straight hairstyles or Black women with straight hairstyles [5]. Furthermore, as shown in recent studies, relaxers and chemical straightening products used to achieve straight hairstyles may contain harmful chemicals that may have detrimental health impacts [6,7,8].

These straightening products—like many other hair products and other personal care products—often contain endocrine disrupting chemicals (EDCs) and carcinogens that have been linked to several health issues including fertility, fibroids, and breast cancer [6,9,10,11,12]. Breast cancer is the leading cause of death for women in the U.S., and Black women are diagnosed at a younger age and have higher mortality rates than other racial groups [13]. EDCs can block normal receptors, act as antagonists, and interfere with normal hormonal processes [11,14]. Parabens, a known EDC, have been shown to alter estrogen receptor target gene expression and increase the viability of genetically diverse luminal breast cancer cell lines [12]. These chemicals found in hair products have also been associated with worse breast tumor clinicopathology [6]. Yet very few interventions are available to reduce these chemical exposures throughout the life course of Black women, who historically use these products more than [7,15] Latina/e, Asian, White, and mixed-race individuals [16,17,18].

Furthermore, there are insufficient tools that examine behaviors and attitudes about perceptions of product use, perceived safety, and breast cancer risk that center on the Black experience of women in the United States. The Study of Environment, Lifestyle, and Fibroids, a prospective study of reproductive-age Black women, used questionnaires to assess the use of personal care products [19], including a variety of hair products [20], but did not collect data on motivations for use, preferences, or potential health concerns related to use. Llanos and colleagues developed The Personal Care Product (PCP) Use questionnaire to assess product use and frequency, which also included seven questions on safety concerns from ingredients in PCPs [16]. Their findings suggest participants have some awareness of the health effects of PCPs and, as a result, reported avoiding certain products. Their sample, however, only included 10% of Black participants. The Taking Stock study and project CAPABLE (Chemical and personal care: Asian, Black and Latina Exposure) collaboratives used a community-based participatory approach to develop and disseminate their tools on personal care product usage [17,18]. However, only information on use among diverse women in California was provided, not the safety concerns, behaviors, or attitudes of participants. The Bankhead/Johnson hair esteem scale includes 10 items used to determine the relationship between hair, self-esteem, and discrimination experiences of Black women [1]. While the tool can be used to explore the attitudes of Black women on wearing their hair in its natural state, safety concerns related to EDCs in hair products are not measured.

Therefore, the Black Identity, Hair Product Use, and Breast Cancer Scale (BHBS) was developed and validated among Black (African American, African, and Caribbean) women aged 29–64 from Southern California to measure the sociocultural perspectives related to their hair, personal care product usage, and breast cancer risk [21]. However, one limitation of the previous BHBS validation study was the lack of representation of Black women with a history of breast cancer. Black breast cancer survivors may face unique challenges with hair loss and identity or satisfaction with appearance because of cancer treatment [22]. In addition, breast cancer survivors may experience greater concerns about cancer risk and/or a greater burden of health-related anxiety [23]. Thus, the purpose of this study was to validate the BHBS in a population of breast cancer survivors.

This study also contributes to the growing literature in exposure science within the context of survivorship that is grounded in the environmental injustice of beauty framework [24]. This framework recognizes the intersecting identities navigated by Black women, including social and economic determinants of health. These factors partially explain Black women’s use of personal care products containing harmful chemicals (e.g., EDCs) to conform to racialized beauty standards rooted in Eurocentric ideals.

## 2. Methods

Participants and Procedures. Women 18 years of age or older who self-identify as Black, African American, African, or Caribbean were recruited from the *Bench to Community Initiative* (BCI), a community-based participatory research program [25]. Data were collected between 22 November 2021 and 22 June 2022, using purposive convenience sampling and snowballing techniques primarily through social media (e.g., Twitter, Facebook, Instagram, and YouTube). The social media channels were created during the COVID-19 pandemic to engage and recruit study participants. Instagram and Facebook accounts using the username *@bench2community* have the most followers. The research team along with BCI community advisory board members and community advocates also posted approved flyers and messaging to recruit study participants via Facebook, Instagram, and Twitter. Additionally, *Mailchimp* was used for targeted email recruitment of individuals from the BCI email listserv who participated in educational forums known as *Salon Conversations* on EDCs and health outcomes hosted by community advisory board members via Zoom Teleconferencing. These *Salon Conversations* were later stored on the BCI YouTube channel. The participants from the BCI project completed a 41-item anonymous survey and received a gift card to purchase non-toxic products through the Black and Green marketplace—a website dedicated to featuring safer, sustainable, Black-owned personal care products [26]. Included in the BCI survey were 27 items on hair identity, product use, and perceived breast cancer risk, including the 11 items that were previously validated as the BHBS. Demographic measures were also collected during this study. 

Demographic measures. Race and ethnicity were assessed using the question “what is your racial affiliation? Please check all that apply” with response options *African, African American, Asian, Caribbean, Central/South American, White, Multiracial*, and *other.* Only individuals that identified as African American, African, and Caribbean were included in the analysis. “What is your age” was an open-ended question used to determine the age of respondents. Breast cancer survivorship status was assessed using the question “have you ever been diagnosed with breast cancer?”, with response options *yes* or *no*. Age of breast cancer diagnosis was an open-ended question (i.e., “age of diagnosis?”). Family history of breast cancer was assessed using the question “have any of your family members been diagnosed with breast cancer?”, with response options *yes* or *no*. Education was measured using the question “what is the highest level of education you have completed?”, with response options *some high school, high school diploma (or equivalent), some college, college degree, graduate degree,* and *professional certification*. Household income was assessed using the question “please select the range of income that is closest to your own”, with response options *less than USD 25,000, USD 26,000–USD 50,000, USD 51,000–USD 75,000, USD 76,000–USD 100,000, USD 100,000–USD 150,000, and more than USD 151,000.* Insurance status was assessed using the question, “do you have health insurance?”, with response options *yes* or *no*. Birthplace was assessed using the question “were you born in the United States?”, with response options *yes* or *no*.

The Black Identity, Hair Product Use, and Breast Cancer Scale (BHBS) measure. This 11-item tool was developed to better understand the sociocultural factors associated with Black identity and perceived breast cancer risk related to toxic chemical exposures in personal care products. The BHBS includes two subscales: *sociocultural perspectives about hair and identity* (S1: 5 items) and *perceived breast cancer risk related to hair product use and selection* (S2: 6 items). S1 questions include (1) Black men do not like Black women to wear their hair natural. (2) In order to be successful in business, it is necessary for Black women to have their hair straight. (3) In order for Black women to attract Black men, they need to straighten their hair. (4) Black women feel pressure from their female friends to straighten their hair. (5) Black women feel pressure from their partners to straighten their hair. S2 questions include (1) I am concerned that the labels of hair care products do NOT list all the ingredients. (2) Because I am concerned about breast cancer, I plan to go natural (style my hair without chemicals). (3) Because I am concerned about breast cancer, I intend to watch the ingredients of the products I will use. (4) All Black women should worry about the ingredients in hair products. (5) Because I am concerned about breast cancer, I plan to adjust how I use hair care products. (6) I want to learn more about the risk hair products can cause to my health. Response options for both subscales are *Strongly Disagree, Disagree, Agree, and Strongly Agree*.

Data Analysis. For this validation study, the same methods were followed as in the prior research to examine the factor structure of the BHBS [21]. Descriptive statistics, including age, age of breast cancer diagnosis, stage of diagnosis, family history of breast cancer, education, household income, insurance status, and birthplace, were examined using SPSS 29.0 [27]. In addition, to investigate the underlying structure and dimensions of the 27 items, a Principal Component Analysis (PCA) was also conducted using SPSS 29.0. Several criteria were used to examine the number and composition of items in each component, including a scree plot of Eigenvalues [28], the Kaiser criterion [29], Horn’s parallel analysis [30], and factor loadings [31]. The criterion cutoff was set at ±0.35 for the item loadings. Based on this criterion, each item loaded most highly on one of two distinct components, with many items loading poorly on additional components. Items that loaded poorly were dropped, and models were reassessed each time by calculating the inter-item reliability of the items for each component based on Cronbach’s alpha (α), which indexes internal scale consistency or the extent to which a scale measures an underlying construct [32].

Confirmatory factor analysis (CFA) was then performed using Mplus software version 8.1 [33] to establish the factor solution that best explained the constructs underlying the Black Identity, Hair Product Use, and Breast Cancer Scale. Based on the initial PCA results, two- and three-factor solutions were examined and compared to one another. The goodness of fit was determined by several indices: chi-square (χ^2^), Akaike’s Information Criterion (AIC), a Root Mean Square Error of Approximation (RMSEA) of <0.10, a Comparative Fit Index CFI of >0.90, and a Tucker Lewis Index (TLI) of >0.90 [34]. The χ^2^ test assesses the overall fit and the discrepancy between the sample and fitted covariance matrices. In addition to the *χ*^2^ test, the AIC is compared for each fitted model [35]. The AIC is a widely used index of fit, such that smaller AIC values are indicative of the most parsimonious and well-fitting model.

## 3. Results

The Black women in this study were educated, with most individuals having obtained college (31%) or graduate (21%) degrees, but frequently reported less than USD 50,000 household income. Most respondents also reported having insurance (93%). Of those who responded regarding the stage of diagnosis (*n* = 71), 19 participants (26%) were Stage 0, 32 participants (45%) were Stage 1, 17 participants (24%) were Stage 2, and 3 participants (4%) were Stage 3. The average age at diagnosis was 37.41 ± 8.8. See Table 1 for a detailed description of the demographic characteristics of the participants.

PCA and CFA. Aligned with previous findings [21], PCA with Varimax rotation yielded two factors that accounted for 63% of the total variance, with 11 of the 27 items loading highly on these two distinct components. The remaining 16 items were dropped due to poor fit (<±0.35), which led to an improvement in internal scale consistency. See Table 2 for the means, standard deviations, skewness, and kurtosis of the BHBS and Appendix A for the initial 27 items.

**Figure 1:**
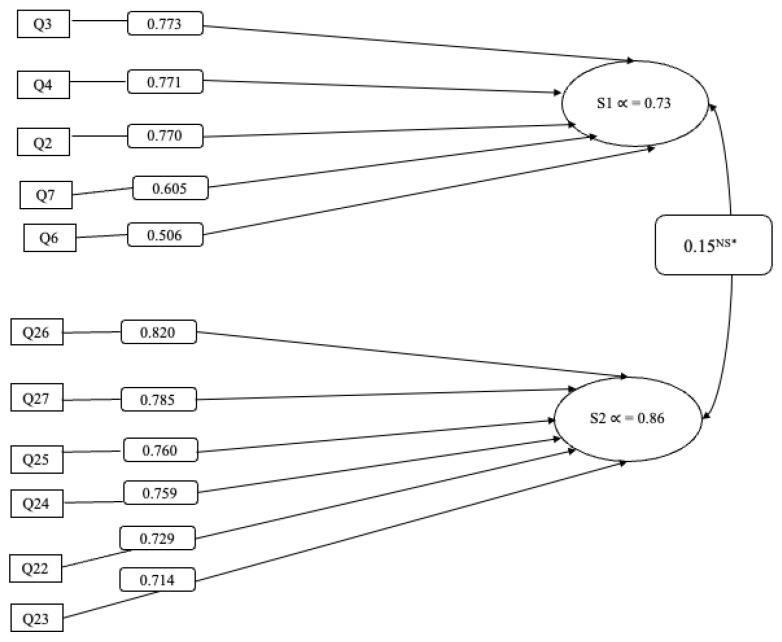
Factor diagram of the 11-item Black Identity, Hair Product Use, and Breast Cancer Scale. Subscale 1 (S1): Sociocultural perspective about hair and identity [Questions: 2, 3, 4, 6, 7]. Subscale 2 (S2): Perceived breast cancer risk related to hair product use [Questions: 22–27]. * NS: Not statistically significant.

Five items measuring *sociocultural perspectives about hair and identity* (subscale 1, S1) accounted for 28% of the total variance (α = 0.73; 95% CI = 0.71–0.82). Six items assessing *perceived breast cancer risk related to hair product use* (subscale 2, S2) accounted for 35% of the total variance (α = 0.86 (95% CI = 0.81–0.94). CFA confirmed the two-component structure (Root Mean Square Error of Approximation = 0.034; Comparative Fit Index = 0.93; Tucker Lewis Index = 0.89). Table 3 presents loadings from PCA for the two factors and Figure 1 includes the factor diagram of the 11-item BHBS.

## 4. Discussion

The BHBS is a valid measure of sociocultural constructs associated with Black women’s hair, identity, and perceived breast cancer risk [21]. The results of this study show that the tool is also valid for Black women who have had a breast cancer diagnosis [21]. Most participants in the current study of Black breast cancer survivors were diagnosed before age 40 and had no family history of breast cancer. The breast cancer incidence among young women (<50 years) in the United States is increasing but varies by race and ethnicity, with a higher prevalence of early-onset breast cancer among Black women compared to White women [13]. This population could benefit from interventions, including earlier screening interventions [36] and reductions in chemical exposures from the use of hair and other personal care products. Additionally, the participants in the present study had comparable demographic characteristics to the U.S. population related to college education (34.3%), insurance status (90.7%), and household income (median USD 75,149) [37].

The validation of the two subscales of the BHBS is consistent with the original study [21] measuring the *sociocultural perspectives about hair and identity* (Subscale 1) and *perceived breast cancer risk related to hair product use* (Subscale 2). Therefore, the BHBS tool can be used to contextualize the experiences related to hair, identity, and perceived breast cancer risk of Black women with or without a breast cancer diagnosis. So, as researchers continue to explore the impact of hair product use on breast cancer risk, it is also appropriate to include sociocultural factors related to identity, product selection behaviors, attitudes, and perceived health risks for Black women. Our findings further suggest that, as interventions to address personal care product use among Black women are developed and tested, Black breast cancer survivors should be included in those studies.

While the sociocultural factors that could be measured using the BHBS tool are important, research is warranted on the hierarchy of competing interests and responsibilities that Black women face during their cancer survivorship journeys. Their survivorship journeys are complex and include unmet physiological, psychological, and spiritual needs that impact Black women’s quality of life (QOL) [38,39,40,41]. Hair loss and/or regrowth after treatment is also of concern for Black women as the lack of hair can be linked to feminine expressions of beauty and acceptance [22]. However, the significance of hair loss and QOL impacts through the lens of Black breast cancer survivorship is limited. As described by Wilson [22], despite Hollywood’s popularization of the *Dora Milaje* in *Wakanda Forever*, baldness in our society is associated with gendered masculinity, unattractiveness, or illness. Black women’s connection to their hair is so significant that loss during treatment can be more distressing than a mastectomy, as hair loss during treatment can be more visible than the loss of one’s breasts [22]. In our previous studies on Black women with or without a history of breast cancer and their partners, we learned the importance of hair also has deep-rooted cultural and societal implications that may supersede the safety of hair products that contain toxic chemicals including EDCs and their potential health consequences [42,43]. Eurocentric or westernized standards of attractiveness that include long straight hair [22] and lighter skin [44] are undoubtedly rooted in slavery, colonization, and other historical contexts, yet these sociocultural perspectives are lacking in discussions related to chemical exposures from personal care products and related breast cancer risk.

Recent studies on personal care product use and EDC exposures have noted differential usage by socioeconomic status [19], neighborhood, and economic variability in the availability of safer products [45], yet the attitudes of participants were not quantified, nor did the findings include breast cancer survivorship experiences. In a qualitative study of women of color in Northern Manhattan, researchers developed an online-delivered environmental health literacy intervention and evaluated the hair journeys of the participants [46]. Cultural integration, described as the impact of one’s culture on behavior, determined the hair care practices of the participants. While none of the participants, except one facilitator, was a breast cancer survivor, researchers also noted time challenges inherent in qualitative data collection efforts. To our knowledge, the BHBS remains the only tool developed to examine intersections of behaviors and attitudes about perceptions of hair, identity, product use, perceived safety, and breast cancer risk for Black women in the United States.

EDCs, such as bisphenol A, phthalates, and parabens, impact the normal estrogen receptor signaling of cells [47]. In a recent study, researchers denoted protumorigenic effects of parabens (e.g., methyl, propyl, and butyl paraben) on West African ancestry luminal breast cancer cell lines [12]. These chemicals are found in personal care products such as straighteners, relaxers, hair dyes, and detanglers, which is of concern to consumers including cancer survivors [14,42]. Therefore, to develop culturally tailored interventions that support the survivorship journeys of Black breast cancer survivors, which includes their hair and hair care practices, we must understand their unmet needs using qualitative and quantitative methods that include validated tools like the BHBS.

Strengths and Limitations. This validation of the BHBS in a survivorship sample should be considered in the context of the following strengths and limitations. The study sample shares demographic similarities to the U.S. population. However, the convenience sampling recruitment methods used may be vulnerable to selection bias. The cross-sectional design and self-reported biases are also notable limitations inherent in the methods of this study. Therefore, our findings cannot be generalized to all Black women or Black breast cancer survivors. The original BHBS validation study was completed in Southern California. While we partnered with community-based organizations throughout the United States and research flyers were shared through various social media channels, we did not collect zip code information and therefore are unable to provide geographical context for findings. Despite these limitations, the BHBS is a validated tool that can be used to ascertain contextual factors related to hair, identity, product use, and breast cancer risk for Black women. The tool can be used in observational and intervention studies to better understand the unmet sociocultural needs of Black breast cancer survivors.

## 5. Conclusions

The BHBS measures sociocultural perceptions about hair, identity, and perceived risk of breast cancer for Black women. These perspectives are important influences on Black women’s survivorship journeys and should be reflected in quality of life interventions. Hair is important to the survivorship journey of Black breast cancer survivors and their identity. They have concerns about EDCs in their personal care products and potential health consequences that remain unaddressed. As emerging research on the impacts of harmful chemicals in personal care products including EDC exposures and breast cancer increases, measuring sociocultural perspectives of Black women should also be considered to meet the needs of this population more fully.

## Figures and Tables

**Table 1:** Demographic characteristics of the participants (*n* = 167).

Sociodemographic Variables	Breast Cancer Survivors *n* (%) *
Race and ethnicity	
African American	162 (97%)
African or Caribbean	5 (3%)
Age, years	
≤29	19 (11%)
30–39	43 (26%)
40–49	59 (35%)
50–59	27 (16%)
>60	19 (11%)
Age at breast cancer diagnosis, years	
≤39	83 (52%)
≥40	77 (48%)
Stage at diagnosis	
Stage 0	19 (26%)
Stage 1	32 (45%)
Stage 2	17 (24%)
Stage 3	3 (4%)
Family history of breast cancer	
Yes	58 (35%)
No	109 (65%)
Education	
≤Some college	69 (41%)
College degree	52 (31%)
Graduate Degree	35 (21%)
Professional Certification	11 (7%)
Household income	
<USD 25,000	14 (9%)
USD 26,000–50,000	57 (35%)
USD 51,000-75,000	49 (30%)
USD 76,000–100,000	18 (11%)
USD 100,000–150,000	14 (9%)
>USD 151,000	12 (7%)
Insurance status	
Yes	155 (93%)
No	11 (7%)
Birthplace	
U.S.	150 (93%)
Not in the U.S.	12 (7%)

* Percentages and number of participants do not equal totals due to missing data.

**Table 2:** Means, standard deviations (SDs), skewness, and kurtosis for the 11 items of the Black Identity, Hair Product Use, and Breast Cancer (BHBS) Scale.

Question No.	BHBS Scale Items	Mean (SD)	Skewness (Kurtosis)
Q2	Black men do not like Black women to wear their hair natural.	2.39 (0.826)	0.324 (−0.362)
Q3	In order to be successful in business, it is a necessary for Black women to have their hair straight.	2.32 (0.886)	0.343 (−0.526)
Q4	In order for Black women to attract Black men, they need to straighten their hair.	2.21 (0.936)	0.489 (−0.549)
Q6	Black women feel pressure from their female friends to straighten their hair.	2.29 (0.838)	0.416 (−0.274)
Q7	Black women feel pressure from their partners to straighten their hair.	2.33 (0.889)	0.211 (−0.646)
Q22	I am concerned that the labels of hair care products do NOT list all the ingredients.	2.98 (0.795)	−0.182 (−0.873)
Q23	Because I am concerned about breast cancer, I plan to go natural (style my hair without chemicals).	2.79 (0.857)	−0.094 (−0.792)
Q24	Because I am concerned about breast cancer, I intend to watch the ingredients of the products I will use.	2.96 (0.808)	−0.345(−0.490)
Q25	All Black women should worry about the ingredients in hair products.	3.05 (0.804)	−0.405 (−0.564)
Q26	Because I am concerned about breast cancer, I plan to adjust how I use hair care products.	2.93 (0.835)	−0.328 (−0.572)
Q27	I want to learn more about the risk hair products can cause to my health.	3.15 (0.819)	−0.671 (−0.178)

Note: Subscale 1: Sociocultural perspective about hair and identity [Questions: 2, 3, 4, 6, 7]. Subscale 2: Perceived breast cancer risk related to hair product use [Questions: 22–27].

**Table 3:** Loadings from PCA for two extracted subscales for the 11-item Black Identity, Hair Product Use, and Breast Cancer (BHBS) Scale.

Question No.	BHBS Scale Items	S1	S2
Q2	Black men do not like Black women to wear their hair natural.	0.770	−0.067
Q3	In order to be successful in business, it is a necessary for Black women to have their hair straight.	0.773	−0.083
Q4	In order for Black women to attract Black men, they need to straighten their hair.	0.771	−0.060
Q6	Black women feel pressure from their female friends to straighten their hair.	0.506	0.014
Q7	Black women feel pressure from their partners to straighten their hair.	0.605	−0.198
Q22	I am concerned that the labels of hair care products do NOT list all the ingredients.	−0.154	0.729
Q23	Because I am concerned about breast cancer, I plan to go natural (style my hair without chemicals).	0.167	0.714
Q24	Because I am concerned about breast cancer, I intend to watch the ingredients of the products I will use.	−0.128	0.759
Q25	All Black women should worry about the ingredients in hair products.	−0.157	0.760
Q26	Because I am concerned about breast cancer, I plan to adjust how I use hair care products.	−0.034	0.820
Q27	I want to learn more about the risk hair products can cause to my health.	−0.181	0.785

Subscale 1 (S1): Sociocultural perspective about hair and identity [Questions: 2, 3, 4, 6, 7]. Subscale 2 (S2): Perceived breast cancer risk related to hair product use [Questions: 22–27].

## Data Availability

The data presented in this are available on request from the corresponding author due to all relevant data are within the manuscript.

## Appendix A

Means, Standard Deviations (SD), Skewness, and Kurtosis for the initial 27 items of the Black identity, hair product use, and breast cancer scale.

	**Questions**	**Mean (SD)**	**Skewness (Kurtosis)**
Q1	Hair has a special role in Black culture.	3.25 (0.797)	−0.693 (−0.424)
**Q2**	**Black men do not like Black women to wear their hair natural**.	**2.39 (0.826)**	**0.324 (−0.362)**
**Q3**	**In order to be successful in business, it is a necessary for Black women to have** **their hair straight**.	**2.32 (0.886)**	**0.343 (−0.526)**
**Q4**	**In order for Black women to attract Black men, they need to straighten their hair**.	**2.21 (0.936)**	**0.489 (−0.549)**
Q5	If I had a daughter, I would want them to straighten their hair.	2.04 (0.827)	0.420 (−0.401)
**Q6**	**Black women feel pressure from their female friends to straighten their hair**.	**2.29 (0.838)**	**0.416 (−0.274)**
**Q7**	**Black women feel pressure from their partners to straighten their hair**.	**2.33 (0.889)**	**0.211 (−0.646)**
Q8	White people are intimidated when they see Black hair in its natural state.	2.68 (0.893)	−0.168 (−0.697)
Q9	Keeping up with current hairstyles is important in Black culture.	2.70 (0.890)	−0.240 (−0.637)
Q10	My hair is a cultural reflection of who I am.	2.87 (0.777)	−0.349 (−0.153)
Q11	Older members of my family would disapprove if I wore my hair natural.	2.26 (0.945)	0.376 (−0.702)
Q12	I believe natural hair can be professional as well as straightened hair.	3.11 (0.924)	−0.565(−0.859)
Q13	Black women should not be concerned about their hair in professional settings.	2.60 (0.864)	−0.054 (−0.634)
Q14	In the work setting, I get treated differently based on how my hair is styled.	2.50 (0.808)	0.226 (−0.440)
Q15	I do not care how much I spend on hair products.	2.28 (0.819)	0.549 (−0.041)
Q16	I do not think chemically altered (relaxer, perm, texturizer, color) hair is harmful.	2.17 (0.943)	0.445 (.650)
Q17	I am completely satisfied with my current hairstyle.	2.83 (0.909)	−0.314 (−0.724)
Q18	I believe natural hair is healthier than chemically altered.	3.11 (0.912)	−0.663 (−0.550)
Q19	Black women spend too much money on hair products.	2.67 (0.864)	0.015 (−0.742)
Q20	I believe there is enough information about breast cancer risk and hair products for Black women.	2.10 (0.935)	0.404 (−0.774)
Q21	I would be interested in education regarding the potential risk between hair products and breast cancer.	3.14 (0.769)	−0.614 (−0.008)
**Q22**	**I am concerned that the labels of hair care products do NOT list all the ingredients**.	**2.98 (0.795)**	**−0.182 (−0.873)**
**Q23**	**Because I am concerned about breast cancer, I plan to go natural (style my hair without chemicals)**.	**2.79 (0.857)**	**−0.094 (−0.792)**
**Q24**	**Because I am concerned about breast cancer, I intend to watch the ingredients of the products I will use**.	**2.96 (0.808)**	**−0.345(−0.490)**
**Q25**	**All Black women should worry about the ingredients in hair products**.	**3.05 (0.804)**	**−0.405 (−0.564)**
**Q26**	**Because I am concerned about breast cancer, I plan to adjust how I use hair care products**.	**2.93 (0.835)**	**−0.328 (−0.572)**
**Q27**	**I want to learn more about the risk hair products can cause to my health**.	**3.15 (0.819)**	**−0.671 (−0.178)**
Note: 11-item Black identity, hair product use and breast cancer scale questions are bolded. Subscale 1: Sociocultural perspective about hair and identity [Questions: 2–4, 6, 7]. Subscale 2: Perceived breast cancer risk related to hair product use [Questions: 22–27].			
